# Supplementary material for: Post-copulatory genetic matchmaking: HLA-dependent effects of cervical mucus on human sperm function
Source: Proc Biol Sci. 2020 Aug 19;287(1933):20201682. doi: 10.1098/rspb.2020.1682 (PMC7482290; doi:10.1098/rspb.2020.1682)
Supplement: Model selection based on AIC values [file rspb20201682supp1.pdf]

Electronic Supplementary Material, Datafile S1:

AIC estimates of full models and final (selected) models

Post-copulatory genetic matchmaking: HLA-dependent effects of cervical mucus on human sperm function

Annalaura Jokiniemi, Martina Magris, Jarmo Ritari, Liisa Kuusipalo, Tuulia Lundgren, Jukka Partanen & Jukka Kekäläinen

Proceedings of the Royal Society B (DOI: 10.1098/rspb.2020.1682)

1. Overall models for the effect of HLA-dissimilarity on sperm swimming velocity (VCL)

<u>Model 5A: Full model with Timepoint * HLA dissimilarity interaction</u>

Model_5A = lmer (VCL ~ 1 + Timepoint * HLA_dissimilarity + (Timepoint|Sub-sample) + (Timepoint|Sub-sample:Replicate) + (Timepoint|Male) + (Timepoint|Female) + (Timepoint|Male:Female), data = All_timepoints)
AIC = 6965.6

<u>Model 5B: Like Model 5A, but without Timepoint * HLA dissimilarity interaction</u>

Model_5B = lmer (VCL ~ 1 + Timepoint + HLA_dissimilarity + (Timepoint|Sub-sample) + (Timepoint|Sub-sample:Replicate) + (Timepoint|Male) + (Timepoint|Female) + (Timepoint|Male:Female), data = All_timepoints)
AIC = 6965.4 (Final model)

2. Overall models for the effect of Genomic dissimilarity on sperm swimming velocity (VCL)

<u>Model 6A: Full model with Timepoint * Genomic dissimilarity interaction</u>

Model_6A = lmer (VCL ~ 1 + Timepoint * Genomic_dissimilarity + (Timepoint|Sub-sample) + (Timepoint|Sub-sample:Replicate) + (Timepoint|Male) + (Timepoint|Female) + (Timepoint|Male:Female), data = All_timepoints)
AIC = 6929.6

<u>Model 6B: Like Model 6A, but random slope, (Timepoint|Sub-sample:replicate), replaced with random intercept, (1|Sub-sample:replicate)</u>

Model_6B = lmer (VCL ~ 1 + Timepoint * Genomic_dissimilarity + (Timepoint|Sub-sample) + (1|Sub-sample:Replicate) + (Timepoint|Male) + (Timepoint|Female) + (Timepoint|Male:Female), data = All_timepoints)
AIC = 6919.6 (Final model)

<u>Model 6C: Like Model 6B, but without Timepoint * Genomic dissimilarity interaction</u>

Model_6C = lmer (VCL ~ 1 + Timepoint + Genomic_dissimilarity + (Timepoint|Sub-sample) + (1|Sub-sample:Replicate) + (Timepoint|Male) + (Timepoint|Female) + (Timepoint|Male:Female), data = All_timepoints)
AIC = 6941.7

3. Overall models for the effect of HLA-dissimilarity on sperm hyperactivation

Model 2A: Full model with Timepoint * HLA dissimilarity interaction

Model_2A = lmer (Hyperactivation ~ 1 + Timepoint * HLA_dissimilarity + (Timepoint|Sub-sample) + (Timepoint|Sub-sample:Replicate) + (Timepoint|Male) + (Timepoint|Female) + (Timepoint|Male:Female), data = All_timepoints)
AIC = 5651.3

Model 2B: Like Model 2A, but without Timepoint * HLA dissimilarity interaction

Model_2B = lmer (Hyperactivation ~ 1 + Timepoint + HLA_dissimilarity + (Timepoint|Sub-sample) + (Timepoint|Sub-sample:Replicate) + (Timepoint|Male) + (Timepoint|Female) + (Timepoint|Male:Female), data = All_timepoints)
AIC = 5647.7 (Final model)

4. Overall models for the effect of Genomic dissimilarity on sperm hyperactivation

Model 3A: Full model with Timepoint * Genomic dissimilarity interaction

Model_3A = lmer (Hyperactivation ~ 1 + Timepoint * Genomic_dissimilarity + (Timepoint|Sub-sample) + (Timepoint|Sub-sample:Replicate) + (Timepoint|Male) + (Timepoint|Female) + (Timepoint|Male:Female), data = All_timepoints)
AIC = 5616.9

Model 3B: Like Model 3A, but random slope, (Timepoint|Sub-sample:replicate), replaced with random intercept, (1|Sub-sample:replicate)

Model_3B = lmer (Hyperactivation ~ 1 + Timepoint * Genomic_dissimilarity + (Timepoint|Sub-sample) + (1|Sub-sample:Replicate) + (Timepoint|Male) + (Timepoint|Female) + (Timepoint|Male:Female), data = All_timepoints)
AIC = 5607.1 (Final model)

Model 3C: Like Model 3B, but without Timepoint * Genomic dissimilarity interaction

Model_3C = lmer (Hyperactivation ~ 1 + Timepoint + Genomic_dissimilarity + (Timepoint|Sub-sample) + (1|Sub-sample:Replicate) + (Timepoint|Male) + (Timepoint|Female) + (Timepoint|Male:Female), data = All_timepoints)
AIC = 5625.5

5. Overall models for the effect of Grantham distance on sperm swimming velocity (VCL)

Model 4A: Full model with Timepoint * Grantham interaction

Model_4A = lmer (VCL ~ 1 + Timepoint * Grantham + (Timepoint|Sub-sample) + (Timepoint|Sub-sample:Replicate) + + (Timepoint|Male) + (Timepoint|Female) + (Timepoint|Male:Female), data = All_timepoints)
AIC = 6949.8 (Final model)

Model 4B: Like Model 4A, but without Timepoint * Grantham interaction

Model_4B = lmer (VCL ~ 1 + Timepoint + Grantham + (Timepoint|Sub-sample) + (Timepoint|Sub-sample:Replicate) + + (Timepoint|Male) + (Timepoint|Female) + (Timepoint|Male:Female), data = All_timepoints)
AIC = 6962.1

6. Overall models for the effect of Grantham distance on sperm hyperactivation

Model 1A: Full model with Timepoint * Grantham interaction

Model_1A = lmer (Hyperactivation ~ 1 + Timepoint * Grantham + (Timepoint|Sub-sample) + (Timepoint|Sub-sample:Replicate) + (Timepoint|Male) + (Timepoint|Female) + (Timepoint|Male:Female), data = All_timepoints)
AIC = 5634.4 (Final model)

Model 1B: Like Model 1A, but without Timepoint * Grantham interaction

Model_1B = lmer (Hyperactivation ~ 1 + Timepoint + Grantham + (Timepoint|Sub-sample) + (Timepoint|Sub-sample:Replicate) + (Timepoint|Male) + (Timepoint|Female) + (Timepoint|Male:Female) , data = All_timepoints)
AIC = 5643.3

7. The effect of Grantham distance on sperm swimming velocity (VCL) at 180 min

Model 8A: Full model with random slope, (Grantham|male)

Model_8A = lmer (VCL ~ 1 + (1|Sub-sample) + (1|Sub-sample:Replicate) + Grantham + (Grantham|Male) +
+ (1|Male) + (1|Female) + (1|Male:Female), data = Time_180)
AIC = 2377.6

Model 8B: Like Model 8A, but without (1|male)

Model_8B = lmer (VCL ~ 1 + (1|Sub-sample) + (1|Sub-sample:Replicate) + Grantham + (Grantham|Male) +
+ (1|Female) + (1|Male:Female), data = Time_180)
AIC = 2375.6 (Final model)

Model 8C: Like Model 8A, but without (Grantham|male)

Model_8C = lmer (VCL_180 ~ 1 + (1|Sub-sample) + (1|Sub-sample:Replicate) + Grantham + (1|Male) +
(1|Female) + (1|Male:Female), data = Time_180)
AIC = 2382.5

8. The effect of Grantham distance on sperm hyperactivation at 180 min

Model 7A: Full model with random slope, (Grantham|male)

Model_7A = lmer (Hyperactivation ~ 1 + (1|Sub-sample) + (1|Sub-sample:Replicate) + Grantham + (Grantham|Male) +
+ (1|Male) + (1|Female) + (1|Male:Female), data = Time_180)
AIC = 1925.9

Model 7B: Like Model 7A, but without (1|male)

Model_7B = lmer (Hyperactivation ~ 1 + (1|Sub-sample) + (1|Sub-sample:Replicate) + Grantham + (Grantham|Male) +
(1|Female) + (1|Male:Female), data = Time_180)
AIC = 1923.9 (Final model)

Model 7C: Like Model 7A, but without (Grantham|male)

Model_7C = lmer (Hyperactivation ~ 1 + (1|Sub-sample) + (1|Sub-sample:Replicate) + Grantham + (1|Male) +
+ (1|Female) + (1|Male:Female), data = Time_180)
AIC = 1934.2

9. The effect of HLA-dissimilarity on sperm viability

Model 10A: Full model with random slope, (HLA_dissimilarity|male)

Model_10A = lmer (Viability ~ 1 + (1|Sub-sample) + (1|Sub-sample:Replicate) + HLA_dissimilarity + (HLA_dissimilarity|Male) + (1|Male) + (1|Female) + (1|Male:Female), data = Viability)
AIC = 1552.7

Model 10B: Like Model 10A, but without (HLA_dissimilarity|male)

Model_10B = lmer (Viability ~ 1 + (1|Sub-sample) + (1|Sub-sample:Replicate) + HLA_dissimilarity + + (1|Male) + (1|Female) + (1|Male:Female), data = Viability)
AIC = 1551.3 (Final model)

10. The effect of Grantham distance on sperm viability

Model 9A: Full model with random slope, (Grantham|male)

Model_9A = lmer (Viability ~ 1 + (1|Sub-sample) + (1|Sub-sample:Replicate) + Grantham + (Grantham|Male) + + (1|Male) + (1|Female) + (1|Male:Female), data = Viability)
AIC = 1556.0

Model 9B: Like Model 9A, but without (Grantham|male)

Model_9B = lmer (Viability ~ 1 + (1|Sub-sample) + (1|Sub-sample:Replicate) + Grantham + (1|Male) + + (1|Female) + (1|Male:Female), data = Viability)
AIC = 1555.3 (Final model)

11. The effect of Genomic dissimilarity on sperm viability

Model 11A: Full model with random slope, (Genomic_dissimilarity|male)

Model_11A = lmer (Viability ~ 1 + (1|Sub-sample) + (1|Sub-sample:Replicate) + Genomic_dissimilarity + (Genomic_dissimilarity|Male) + (1|Male) + (1|Female) + (1|Male:Female), data = Viability)
AIC = 1559.9

Model 11B: Like Model 11A, but without (Genomic_dissimilarity|male)

Model_11B = lmer (Viability ~ 1 + (1|Sub-sample) + (1|Sub-sample:Replicate) + Genomic_dissimilarity + + (1|Male) + (1|Female) + (1|Male:Female), data = Viability)
AIC = 1554.6 (Final model)
